# High Mobility Group A1 Regulates Transcription Levels of Oligodendrocyte Marker Genes in Cultured Oligodendrocyte Precursor Cells

**DOI:** 10.3390/ijms23042236

**Published:** 2022-02-17

**Authors:** Naohiro Egawa, Gen Hamanaka, Kelly K. Chung, Hidehiro Ishikawa, Akihiro Shindo, Takakuni Maki, Ryosuke Takahashi, Haruhisa Inoue, Eng H. Lo, Ken Arai

**Affiliations:** 1 Neuroprotection Research Laboratory, Departments of Radiology and Neurology, Massachusetts General Hospital and Harvard Medical School, Boston, MA 02129, USA; ghamanaka@mgh.harvard.edu (G.H.); kkchung@mgh.harvard.edu (K.K.C.); hishikawa@mgh.harvard.edu (H.I.); a-shindo@clin.medic.mie-u.ac.jp (A.S.); harutoma@kuhp.kyoto-u.ac.jp (T.M.); lo@helix.mgh.harvard.edu (E.H.L.); 2 Department of Neurology, Graduate School of Medicine, Kyoto University, Kyoto 606-8501, Japan; ryosuket@kuhp.kyoto-u.ac.jp; 3 iPSC-Based Drug Discovery and Development Team, RIKEN BioResource Research Center (BRC), Kyoto 619-0237, Japan; haruhisa@cira.kyoto-u.ac.jp; 4 Center for iPS Cell Research and Application (CiRA), Kyoto University, Kyoto 606-8501, Japan; 5 Medical-Risk Avoidance Based on iPS Cells Team, RIKEN Center for Advanced Intelligence Project (AIP), Kyoto 606-8507, Japan

**Keywords:** cell differentiation, epigenetic regulator, HMGA1, oligodendrocyte precursor cell

## Abstract

Oligodendrocyte precursor cells (OPCs) serve as progenitor cells of terminally differentiated oligodendrocytes. Past studies have confirmed the importance of epigenetic system in OPC differentiation to oligodendrocytes. High mobility group A1 (HMGA1) is a small non-histone nuclear protein that binds DNA and modifies the chromatin conformational state. However, it is still completely unknown about the roles of HMGA1 in the process of OPC differentiation. In this study, we prepared primary OPC cultures from the neonatal rat cortex and examined whether the loss- and gain-of-function of HMGA1 would change the mRNA levels of oligodendrocyte markers, such as *Cnp*, *Mbp*, *Myrf* and *Plp* during the process of OPC differentiation. In our system, the mRNA levels of *Cnp*, *Mbp*, *Myrf* and *Plp* increased depending on the oligodendrocyte maturation step, but the level of *Hmga1* mRNA decreased. When HMGA1 was knocked down by a siRNA approach, the mRNA levels of *Cnp*, *Mbp*, *Myrf* and *Plp* were smaller in OPCs with *Hmga1* siRNA compared to the ones in the control OPCs. On the contrary, when HMGA1 expression was increased by transfection of the *Hmga1* plasmid, the mRNA levels of *Cnp*, *Mbp*, *Myrf* and *Plp* were slightly larger compared to the ones in the control OPCs. These data may suggest that HMGA1 participates in the process of OPC differentiation by regulating the mRNA expression level of myelin-related genes.

## 1. Introduction

The oligodendrocyte precursor cell (OPC) serves as a progenitor cell of terminally differentiated oligodendrocytes. Matured oligodendrocytes form myelin sheaths around axons in the central nervous system, and the myelin sheath is essential in fast impulse propagation through the myelinated fiber. Since oligodendrocytes do not proliferate, OPCs play an essential role in increasing the number of oligodendrocytes during development or after oligodendrocyte/myelin damage. Mechanisms of OPC differentiation have been extensively examined, and several extrinsic signaling molecules have been identified as regulators of OPC differentiation into oligodendrocytes [1,2,3]. In addition, recent studies have confirmed the critical role of the epigenetic system in OPC proliferation and differentiation [4,5,6,7,8,9,10,11,12,13].

DNA chromatin modifications alter the gene expression, a process known as epigenetic regulation. Past studies have demonstrated that epigenetic regulation is closely linked to cell differentiation in most organs, including neurogenesis in the brain [14]. High mobility group A1 (HMGA1) is a small protein that binds DNA and modifies the chromatin state [15,16,17]. Therefore, HMGA1 contributes to the regulation of gene expression by modulating the accessibility of regulatory factors to the DNA. However, no study has ever examined whether and how HMGA1 is involved in oligodendrocyte lineage cells. Therefore, we used a primary OPC culture system to examine whether HMGA1 deficiency or overexpression during the process of OPC differentiation would change the expression patterns of myelin-related genes. Since OPCs play an important role in increasing the number of oligodendrocytes not only during the developmental stage but also after brain injury, as a part of the Special Issue “Molecular Research on Neurodegenerative Diseases 2.0”, we report here that HMGA1 may contribute to OPC differentiation.

## 2. Materials and Methods

All experiments were performed following institutionally approved protocols by the Massachusetts General Hospital Subcommittee on Research Animal Care and in accordance with the National Institutes of Health Guide for the Care and Use of Laboratory Animals.

**Figure 1:**
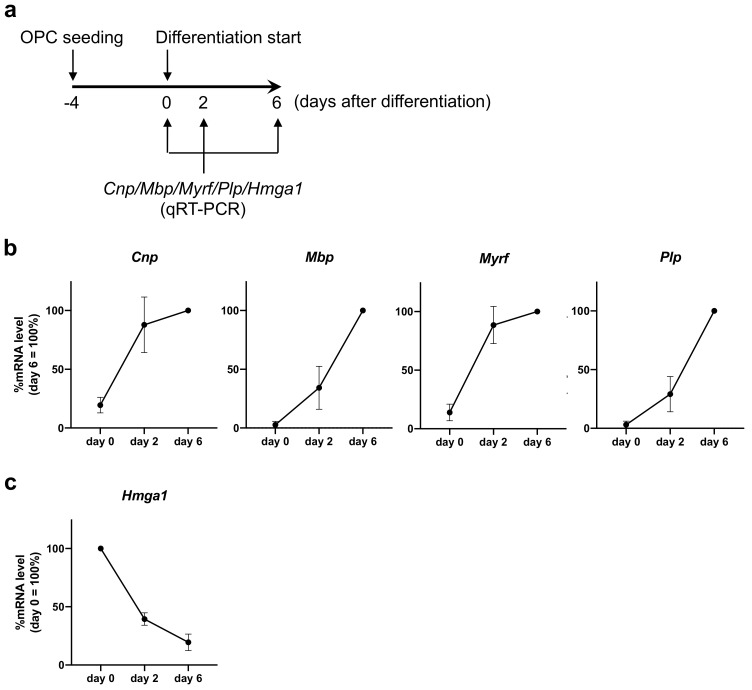
mRNA expression of myelin-related genes and *Hmga1* in an OPC culture. (**a**) A diagram of an experimental schedule. Four or 5 days after OPC seeding (i.e., 70–80% confluency), OPCs started differentiation into oligodendrocytes by switching the culture media from “proliferation media” to “differentiation media”. RNA samples were then prepared at days 0, 2, and 6 after the initiation of OPC differentiation. (**b**) Expression levels of mRNA for *Cnp*, *Mbp*, *Myrf* and *Plp* were all increased by OPC differentiation, as expected. Data are the mean ± SD from 4 independent experiments. (**c**) Expression level of mRNA for *Hmga1* was increased by OPC differentiation. Data are the mean ± SD from 3 independent experiments.

**Figure 2:**
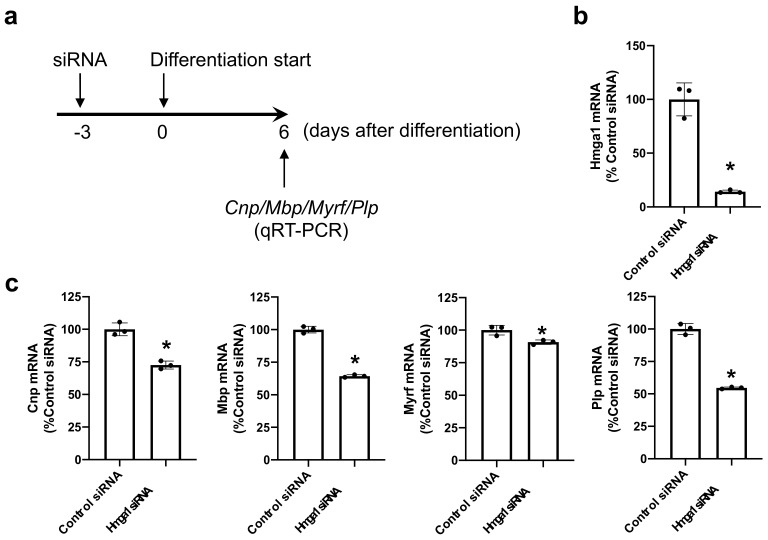
HMGA1 and OPC differentiation (loss-of-function experiments). (**a**) OPCs were transfected with either control siRNA or *Hmga1* siRNA 3 days before the initiation of OPC differentiation. Then, 6 days after OPC differentiation, the cells were collected and subjected to QRT-PCR for assessing the mRNA levels of the myelin-related genes *Cnp*, *Mbp*, *Myrf* and *Plp*. (**b**) OPCs with *Hmga1* siRNA showed a lower mRNA level of *Hmga1* at day 0 of OPC differentiation (e.g., 3 days after the siRNA transfection). Data are the mean ± SD from 3 independent experiments. * *p* < 0.05 vs. the control siRNA group. (**c**) mRNA levels for the myelin-related genes *Cnp*, *Mbp*, *Myrf* and *Plp* were all decreased by *Hmga1* siRNA transfection. Data are the mean ± SD from 3 independent experiments. * *p* < 0.05 vs. the control siRNA group.

**Figure 3:**
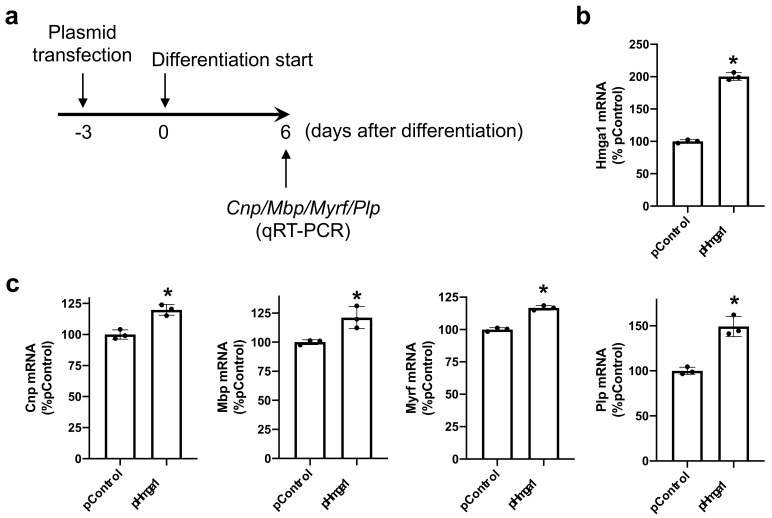
HMGA1 and OPC differentiation (gain-of-function experiments). (**a**) OPCs were transfected with either the control plasmid (pControl) or *Hmga1*-containing plasmid (pHmga1) 3 days before the initiation of OPC differentiation. Then, 6 days after OPC differentiation, the cells were collected and subjected to QRT-PCR for assessing the mRNA levels of the myelin-related genes *Cnp*, *Mbp*, *Myrf* and *Plp*. (**b**) OPCs with *Hmga1*-containing plasmid showed a larger mRNA level of *Hmga1* 2 days after siRNA transfection. Data are the mean ± SD from 3 independent experiments. * *p* < 0.05 vs. the pControl group. (**c**) mRNA levels for the myelin-related genes *Cnp*, *Mbp*, *Myrf* and *Plp* were all increased slightly but significantly by transfection of the *Hmga1*-containing plasmid. Data are the mean ± SD from 3 independent experiments. * *p* < 0.05 vs. the pControl group.

**Figure 4:**
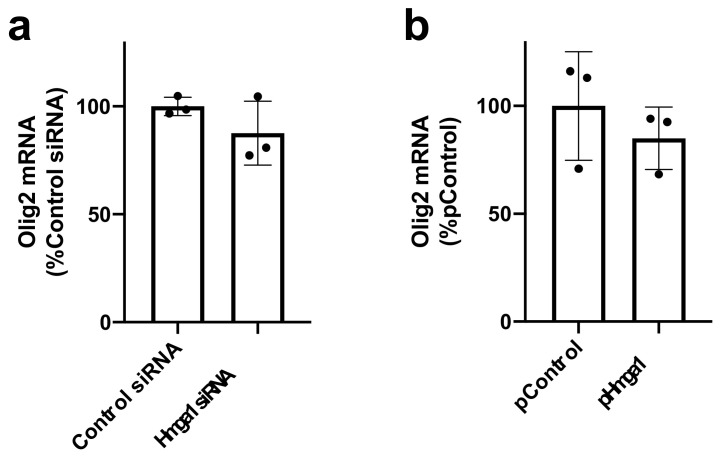
HMGA1 and *Olig2* expression in OPCs. (**a**) OPCs were transfected with either control siRNA or *Hmga1* siRNA 3 days before the initiation of OPC differentiation. Then, 6 days after OPC differentiation, the cells were collected and subjected to QRT-PCR for assessing the mRNA level for *Olig2*, a major transcription factor in oligodendrocyte lineage cells. The mRNA level *Olig2* was not significantly changed by *Hmga1* siRNA transfection. Data are the mean ± SD from 3 independent experiments. (**b**) OPCs were transfected with either control plasmid (pControl) or *Hmga1*-containing plasmid (pHmga1) 3 days before the initiation of OPC differentiation. Then, 6 days after OPC differentiation, the cells were collected and subjected to QRT-PCR for assessing the mRNA level for *Olig2*. mRNA level *Olig2* was not significantly changed by *Hmga1* plasmid transfection. Data are the mean ± SD from 3 independent experiments.

OPC isolation: Primary cortical OPCs were prepared for experiments according to our previous works [13,18,19]. Briefly, primary mixed glial cells were obtained from the brains of postnatal day 1 SD rats and cultured in Dulbecco’s Modified Eagle’s Medium (DMEM; Thermo Fisher Scientific, Waltham, MA, USA) containing 20% fetal bovine serum (ATLANTA Biologicals, Flowery Branch, GA, USA) and 1% of penicillin/streptomycin (P/S, Thermo Fisher Scientific, Waltham, MA, USA). Ten days later, the flasks were shaken for 1 h on an orbital shaker (218 rpm) at 37 °C to remove microglia. They were then changed to a new medium and shaken overnight (~20 h). The medium was collected and plated on noncoated tissue culture dishes for 1 h at 37 °C to remove the contaminated astrocytes and microglia. Then, the nonadherent cells (i.e., OPCs) in the culture media were collected and seeded onto poly-DL-ornithine-coated plates (Sigma, St. Louis, MO, USA). OPC differentiation was initiated on day 4 after cell seeding by changing the culture media from the OPC-proliferating media (neurobasal medium (Thermo Fisher Scientific, Waltham, MA, USA) containing 2% B27 supplement (Thermo Fisher Scientific, Waltham, MA, USA), 1% of P/S, 2 mM glutamine (Thermo Fisher Scientific, Waltham, MA, USA), 10 ng/mL PDGF-AA (PEPROTECH, Rocky Hill, NJ, USA), and 10 ng/mL FGF-2 (PEPROTECH, Rocky Hill, NJ, USA)) to the OPC differentiation media (DMEM containing 2% B27 supplement, 10 ng/mL CNTF (PEPROTECH, Rocky Hill, NJ, USA), and 15 nM T3 (Sigma, St. Louis, MO, USA)). siRNA or plasmid transfection was conducted 1 day (i.e., 3 days before starting OPC differentiation) after OPC seeding. For Figure 1, we conducted each experiment in triplicate. However, the experiments for Figure 2, Figure 3 and Figure 4 were not conducted in triplicate, because our data in Figure 1 suggested that our OPC culture system was stable, and experiments done in triplicate were not necessary for this study.

Transfection with plasmid and siRNA: OPC was transfected with siRNA universal negative control (MISSION, Sigma, St. Louis, MO, USA) or siRNA targeted for specific gene suppression using Lipofectamine 3000 (Thermo Fisher Scientific, Waltham, MA, USA). The pCMV6-*Hmga1*(1-321) vector was generated from the pCMV6 vector (#PS100001, Addgene, Watertown, MA, USA). The pCMV6 vector contained GFP, and the transfection of the pCMV6 vector was validated with GFP signaling.

Quantitative RT-PCR (QRT-PCR): The RNA was isolated with the RNeasy Plus Mini kit (QIAGEN, Hilden, Germany), and first-stranded cDNA was synthesized with the PrimeScript RT reagent (Takara-Clonetech, San Jose, CA, USA). QRT-PCR was performed using SYBR Premix Ex Taq II (Takara-Clonetech, San Jose, CA, USA) and analyzed with the Fast Real-Time System 7500 (Applied Biosystems, Waltham, MA, USA). The expression levels of each target gene were measured relative to GAPDH as the internal control. The following sequences of primers were used: 5′-tccagtatgactctacccacg-3′ for GAPDH forward; 5′-cacgacatactcagcaccag-3′ for GAPDH reverse; 5′-actgccaacaacatgcggaagaag-3′ for *Myrf* forward; 5′-tgggttagaggcccgaacaatgat-3′ for *Myrf* reverse; 5′-ctactttggcaagagacctcc -3′ for CNP forward; 5′-agagatggacagtttgaaggc-3′ for CNP reverse; 5′-ttgactccatcgggcgcttcttta-3′ for MBP forward; 5′-ttcatcttgggtcctctgcgactt-3′ for MBP reverse; 5′-tctttggcgactacaagaccacca-3′ for PLP forward; 5′-caaacaatgacacacccgctccaa-3′ for PLP reverse; 5′-caggaaaaggatgggactga-3′ for *Hmga1* forward; 5′-cagaggactcctgggagatg-3′ for *Hmga1* reverse.

Statistical analysis: The statistical significance was evaluated using the unpaired *t*-test (or Welch’s *t*-test if the normality of distribution was not assumed) to compare differences between the two groups. Data are expressed as the mean ± S.D. A *p*-value of <0.05 was considered statistically significant.

## 3. Results

We first checked if our OPC cultures were functional, i.e., they successfully differentiated into mature oligodendrocytes. We prepared primary OPC cultures from the neonatal rat brain cortex. When we changed the culture media from “Neurobasal plus PDGF-AA and basic FGF (proliferation media)” to “DMEM plus CNTF and T3 (differentiation media)”, as expected, the mRNA levels of myelin-related genes, such as *Cnp*, *Mbp*, *Myrf* and *Plp*, increased over time (Figure 1a,b). Under these conditions, the *Hmga1* mRNA level gradually decreased, depending on the maturation process of the OPCs (Figure 1c).

To examine the roles of HMGA1 in OPC differentiation, we then transfected siRNA for HMGA1 into OPC cultures (Figure 2a). This protocol successfully downregulated the mRNA level of *Hmga1* when the OPCs were initiated for their differentiation (Figure 2b). On day 6 after the initiation of OPC differentiation, cells with *Hmga1* siRNA showed lower mRNA levels of myelin-related genes (Figure 2c). On the contrary, when the OPCs were transfected with the *Hmga1*-containing plasmid, the mRNA level of *Hmga1* was increased (Figure 3a,b), and the mRNA levels of myelin-related genes in the OPCs with *Hmga1* plasmid increased slightly but significantly at day 6 after the initiation of OPC differentiation (Figure 3c).

Finally, we also examined whether HMGA1 also participates in the expression of transcription factors that are specifically expressed in oligodendrocyte lineage cells in the brain. *Olig2* is one of the major transcription factors that regulate the cell fate of oligodendrocyte lineage cells. *Olig2* mRNA was detected in both OPCs and oligodendrocytes in our system, as expected (data not shown). However, a loss-of-function or a gain-of-function experiment demonstrates that HMGA1 may not play a significant contribution to the regulation of *Olig2* expression (Figure 4a,b).

## 4. Discussion

HMGA1 is a small nonhistone protein that does not have transcriptional activity; however, HMGA1 can bind DNA and modify the chromatin conformational state, thus contributing to the modulation of gene expression through changing the accessibility of several regulatory factors to DNA [15,16,17]. In this study, we demonstrate that (i) OPCs express HMGA1, (ii) the expression levels of *Hmga1* mRNA decrease, along with OPC differentiation, and (iii) HMGA1 may regulate the expression levels of some oligodendrocyte marker genes, thus contributing to the process of oligodendrocyte generation. The expression level of HMGA1 is shown to be regulated by Hes5, a transcriptional repressor [20]. During the developmental stage, *Hes5* overexpression downregulated the *Hmga1* expression level in the brain, along with the decrease in the number of oligodendrocytes [20]. In addition, another study showed that, compared to wild-type mice, the expression levels of myelin genes were upregulated in *Hes5* knockout mice [21]. Therefore, our findings support the idea that the process of oligodendrocyte generation is tightly regulated by the epigenetic system and may also fill the knowledge gap about how the *Hes5*–*Hmga1* pathway contributes to the cell fate decision of oligodendrocyte lineage cells.

Oligodendrocytes play an essential role in fast impulse propagation in the central nervous system by generating a myelin sheath that allows the rapid conduction of action potentials along axons. Since oligodendrocytes do not proliferate, OPC proliferation and differentiation into oligodendrocytes are essential steps for a proper central nervous system network during development. Even in the adult brain, OPCs are widely distributed, comprising approximately 5% of all brain cells [22,23,24]. These residual OPCs in the adult brain are relatively quiescent, but after brain damage, they rapidly proliferate and differentiate into mature oligodendrocytes to restore myelin sheaths [25,26,27,28]. The underlying mechanisms of OPC differentiation have been extensively examined, and several molecules, including epigenetic regulators, are identified to promote/suppress OPC differentiation [1,2,3]. However, to our best knowledge, there has been no study that examined the role of HMGA1 in the function of oligodendrocyte lineage cells. Therefore, our current study may help us to understand the detailed molecular regulatory mechanisms of OPC-to-oligodendrocyte differentiation.

Our study is the first to show the involvement of HMGA1 in OPC differentiation, but because of the nature of the Short Communication study, there are some important limitations/caveats in this study, which would be carefully considered for future studies. First, although there are two HMGA proteins (HMGA1 and HMGA2) [15,16,17], we only focused on the roles of HMGA1 in this study. This is partly because the mRNA level of *Hmga1* is much larger (~10 fold) than that of *Hmga2* in our OPC culture system (data not shown). However, we have not examined the changes in the expression pattern of HMGA2 during the process of OPC differentiation. Therefore, it is possible that HMGA2 expression would increase after the initiation of differentiation, and HMGA2 would participate in the maintenance of the process of OPC differentiation. Secondly, we conducted the gain- and loss-of-function experiments under physiological conditions. In general, HMGA1 is abundantly expressed during the developmental stage but is downregulated in adult differentiated tissues [16,17]. This may be consistent with our finding that HMGA1 expression in OPCs is larger than the one in oligodendrocytes. However, after white matter damage, OPCs would be activated to initiate the process of oligodendrogenesis as a compensatory mechanism [29]. Therefore, to pursue the therapeutic potential of HMGA1 for white matter-related diseases, it would be required to examine the expression level of HMGA1 in OPCs under diseased conditions using in vitro and in vivo models. Third, this study specifically asked the question whether HMGA1 regulates the transcriptional level of oligodendrocyte marker genes in cultured OPCs. However, our preliminary experiments of immunostaining also show that *Hmga1* knockdown may reduce the process of OPC maturation in vitro, assessed by GST-pi/MBP staining (Supplementary Figure S1). Therefore, we should take the post-transcriptional protein levels of these markers into consideration to further our understanding of the role of HMGA1 in the oligodendrocyte maturation step. In addition, although we examined the link between HMGA1 and the transcription factor *Olig2*, OPC differentiation is regulated by several transcription factors besides *Olig2*. To reveal the link between HMGA1 and transcriptional regulation, future studies need to check the role of HMGA1 in these transcription factors, such as *Olig1*, *Sox10*, *ID2*, *ID4* and *YY1* [30]. Finally, we have not identified which genes would be specifically targeted by HMGA1. In this study, while *Hmga1* knockdown decreased the mRNA expression levels of myelin-related genes (*Cnp*, *Mbp*, *Myrf* and *Plp*), it did not cause any change in the mRNA level of *Olig2*, which is an important transcription factor for oligodendrocyte lineage cells. Hence, a deeper understanding of the specificity of HMGA1 would help us to understand the physiological and pathological mechanisms of oligodendrocyte (re)generation.

In summary, we demonstrated that HMGA1 regulates the mRNA level of some oligodendrocyte marker genes, supporting the idea that HMGA1 may contribute to the process of OPC differentiation. OPCs play an indispensable role in increasing the number of oligodendrocytes not only during the developmental stage but also after white matter damage in adult brains. Therefore, understanding the roles of HMGA1 in oligodendrocyte lineage cells may help us to find a novel therapeutic approach for white matter-related CNS diseases, such as stroke or vascular dementia.

## Data Availability

The data presented in this study are available on request from the corresponding author.

## Supplementary Materials

The following supporting information can be downloaded at: https://www.mdpi.com/article/10.3390/ijms23042236/s1.
