# Supplementary material for: High Mobility Group A1 Regulates Transcription Levels of Oligodendrocyte Marker Genes in Cultured Oligodendrocyte Precursor Cells
Source: Int J Mol Sci. 2022 Feb 17;23(4):2236. doi: 10.3390/ijms23042236 (PMC8878090; doi:10.3390/ijms23042236)
Supplement: Supplementary file 1 [file ijms-23-02236-s001.zip › ijms-1553161-supplementary.pdf]

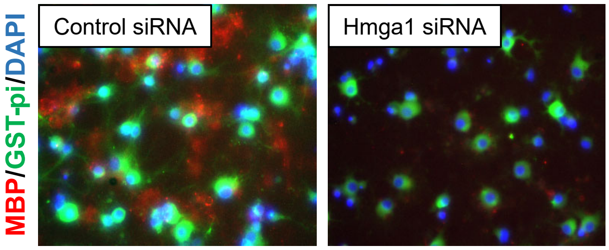

Supplementary Figure S1:

OPCs were transfected with either control siRNA or *Hmga1* siRNA 3 days before the initiation of OPC differentiation. Then, 6 days after OPC differentiation, cells were fixed using 4% PFA for 15 min. After being washed three times in PBS, they were incubated with BLOCK ACE (DS Pharma Biomedical Co., Ltd) for 1 h. Cells were immunostained with primary antibodies of anti-MBP (1:100, Thermo Scientific) and anti-GSTpi (1:200, MBL), which are markers for oligodendrocytes. Experiments were repeated 4 times. The images are representative immunostaining patterns for each group.
